# Supplementary material for: Venomix: a simple bioinformatic pipeline for identifying and characterizing toxin gene candidates from transcriptomic data
Source: PeerJ. 2018 Jul 31;6:e5361. doi: 10.7717/peerj.5361 (PMC6074769; doi:10.7717/peerj.5361)
Supplement: Supplemental Information 3 [file peerj-06-5361-s003.gz › FinalOutput_E-6/C-type_lectin_galatrox_1/finaltree.pdf]

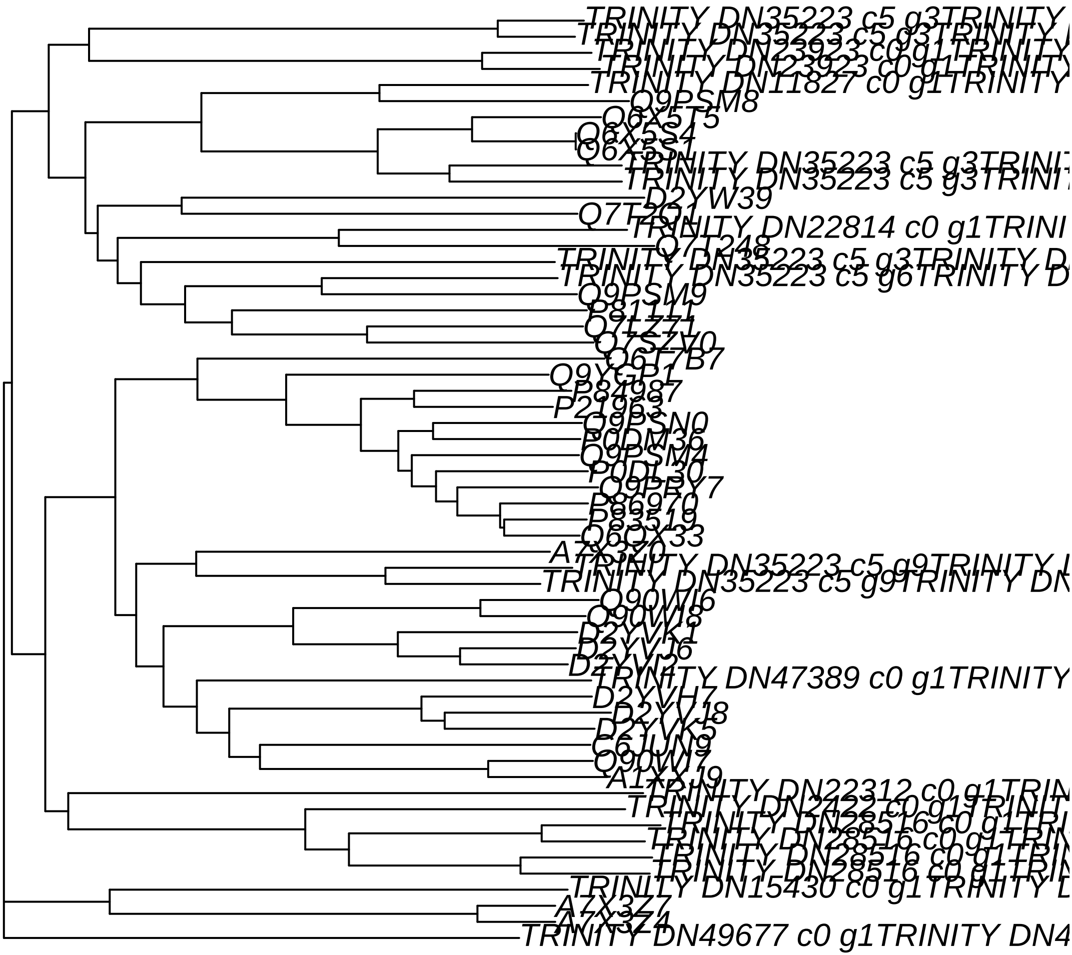
TRINITY DN35223 c5 g3TRINITY
TRINITY DN23923 c0 g1TRINITY
TRINITY DN11827 c0 g1TRINITY
Q9PSM8
Q6X5T5
Q6X5S4
TRINITY DN35223 c5 g3TRINI
D2YW39
TRINITY DN22814 c0 g1TRINI
Q7T248
TRINITY DN35223 c5 g6TRINITY D
Q9PSM9
Q7LZ71
Q7SZV0
Q6T7B7
Q9YGP1
P84987
P21963
Q9PSN0
P0DM36
Q9PSM4
P0DL30
Q9PRY7
P86970
P83519
Q6QX33
A7X3Z0
TRINITY DN35223 c5 g9TRINITY DN
Q90WI6
Q90WI8
D2YVK1
D2YVJ6
TRINITY DN47389 c0 g1TRINITY
D2YVH7
D2YVJ8
D2YVK5
C6JUN9
Q90WI7
A1XXJ9
TRINITY DN22312 c0 g1TRIN
TRINITY DN28516 c0 g1TRIN
TRINITY DN15430 c0 g1TRINITY
A7X3Z7
A7X3Z4
TRINITY DN49677 c0 g1TRINITY DN4
